# Tissue Distribution of Kir7.1 Inwardly Rectifying K^+^ Channel Probed in a Knock-in Mouse Expressing a Haemagglutinin-Tagged Protein

**DOI:** 10.3389/fphys.2018.00428

**Published:** 2018-04-23

**Authors:** Isabel Cornejo, Sandra Villanueva, Johanna Burgos, Karen I. López-Cayuqueo, Régine Chambrey, Francisca Julio-Kalajzić, Neudo Buelvas, María I. Niemeyer, Dulce Figueiras-Fierro, Peter D. Brown, Francisco V. Sepúlveda, L. P. Cid

**Affiliations:** ^1^Centro de Estudios Científicos, Valdivia, Chile; ^2^Universidad Austral de Chile, Valdivia, Chile; ^3^Institut National de la Santé et de la Recherche Médicale, Unité Mixte de Recherche S970, PARCC, Hôpital Européen Georges Pompidou, Assistance Publique Hôpitaux de Paris, Paris, France; ^4^School of Medical Sciences, University of Manchester, Manchester, United Kingdom

**Keywords:** epithelial transport, K^+^ channel, choroid plexus, respiratory epithelium, renal tubule, knock-in mouse, CRISPR-Cas

## Abstract

Kir7.1 encoded by the *Kcnj13* gene in the mouse is an inwardly rectifying K^+^ channel present in epithelia where it shares membrane localization with the Na^+^/K^+^-pump. Further investigations of the localisation and function of Kir7.1 would benefit from the availability of a knockout mouse, but perinatal mortality attributed to cleft palate in the neonate has thwarted this research. To facilitate localisation studies we now use CRISPR/Cas9 technology to generate a knock-in mouse, the Kir7.1-HA that expresses the channel tagged with a haemagglutinin (HA) epitope. The availability of antibodies for the HA epitope allows for application of western blot and immunolocalisation methods using widely available anti-HA antibodies with WT tissues providing unambiguous negative control. We demonstrate that Kir7.1-HA cloned from the choroid plexus of the knock-in mouse has the electrophysiological properties of the native channel, including characteristically large Rb^+^ currents. These large Kir7.1-mediated currents are accompanied by abundant apical membrane Kir7.1-HA immunoreactivity. WT-controlled western blots demonstrate the presence of Kir7.1-HA in the eye and the choroid plexus, trachea and lung, and intestinal epithelium but exclusively in the ileum. In the kidney, and at variance with previous reports in the rat and guinea-pig, Kir7.1-HA is expressed in the inner medulla but not in the cortex or outer medulla. In isolated tubules immunoreactivity was associated with inner medulla collecting ducts but not thin limbs of the loop of Henle. Kir7.1-HA shows basolateral expression in the respiratory tract epithelium from trachea to bronchioli. The channel also appears basolateral in the epithelium of the nasal cavity and nasopharynx in newborn animals. We show that HA-tagged Kir7.1 channel introduced in the mouse by a knock-in procedure has functional properties similar to the native protein and the animal thus generated has clear advantages in localisation studies. It might therefore become a useful tool to unravel Kir7.1 function in the different organs where it is expressed.

## Introduction

The inwardly rectifying Kir7.1 K^+^ channel is encoded by the *Kcnj13* gene in the mouse and is classified as a K^+^-transport type channel. Kir7.1 is the latest addition to the Kir family of proteins (Hibino et al., 2010), and it diverges from classical Kir proteins in sequence and functional properties (Döring et al., 1998; Krapivinsky et al., 1998; Partiseti et al., 1998). Thus, Kir7.1 channels are largely voltage-independent and lack the characteristic Kir K^+^ concentration-dependence.

Kir7.1 is present in epithelial tissues where invariably it has been found to share the same sidedness with the Na^+^/K^+^-pump. Kir7.1 is present at the basolateral membrane of intestinal epithelial cells, thyroid follicular cells and epithelial cells of renal proximal or distal tubules (Nakamura et al., 1999; Ookata et al., 2000; Derst et al., 2001). The Na^+^/K^+^-pump is expressed at a basolateral membrane location in these epithelia as in most transporting barriers. Kir7.1, however, is found at the apical membranes of retinal pigmented epithelium (RPE) and choroid plexus. These epithelia show an unusual apical membrane expression of the Na^+^/K^+^-pump (Nakamura et al., 1999; Kusaka et al., 2001; Shimura et al., 2001; Yang et al., 2003). The faithful distribution of Kir7.1 together with the Na^+^/K^+^-pump has prompted the speculation that Kir7.1’s function might be to provide a pathway to recycle K^+^ taken up pump in exchange for Na^+^ thus maintaining transepithelial ion transport processes without potentially deleterious intracellular K^+^ accumulation (Sepúlveda et al., 2015). Other roles attributed to Kir 7.1 include a participation in the mechanism of melanocortin-mediated regulation of energy homeostasis within the paraventricular nucleus (Ghamari-Langroudi et al., 2015) and the control of excitability of uterine smooth muscle in the transition to contractions in the pregnant uterus (McCloskey et al., 2014).

Diseases associated with mutations in human *KCNJ13* include snowflake vitreoretinal degeneration (SVD) (Hejtmancik et al., 2008) and Leber congenital amaurosis (Sergouniotis et al., 2011), which are caused by trafficking or functional defects (Sepúlveda et al., 2015). A study of a mouse model with mosaic expression of *Kcnj13* in the RPE suggests a role of Kir7.1 in the ionic regulation of the confined space between RPE and photoreceptors possibly in relation to lactate transport (Zhong et al., 2015). Experiments using shRNA knock down of Kir7.1 expression or pharmacological inhibition of the channel also suggest a role for Kir7.1 in the ionic regulation at the RPE/photoreceptor interface (Shahi et al., 2017).

Further investigation into the function of Kir7.1 in the organism would be greatly facilitated by the availability of a *Kcnj13* gene knock-out mouse. Such an animal model would also provide an ideal negative control for studies of protein expression by western blot, immunohistochemistry or immunofluorescence. We have previously generated Kir7.1 null mutant mice (Villanueva et al., 2015), but these animals die hours after birth most probably due to incomplete palate sealing, a malformation known to lead to perinatal mortality (Turgeon and Meloche, 2009). In addition Kir7.1 null mice have a moderate retardation in lung development, which was surprising as there was no evidence for a role of the channel in the respiratory system.

In order to understand better the tissue distribution of Kir7.1 we have now generated a knock-in mouse that expresses the channel tagged with a haemagglutinin (HA) epitope using the CRISPR Cas9 technology. The availability of antibodies for the HA epitope allows for application of western blot and immunolocalisation methods without need for channel-specific reagents. Importantly, WT tissues provide an unequivocal negative control adding robustness to the assays. We check for Kir7.1-HA functional properties, confirm the presence of the channel in well characterized locations and explore its expression in kidney and respiratory epithelium, tissues with controversial or unreported localization respectively.

## Materials and Methods

### Animals

We used C57BL6/J mice from Jackson Laboratories (United States). Mice were maintained on standard laboratory chow (2019S diet, Harlan Laboratories) and water *ad libitum*, under regulated temperature (22–26°C) and light (12:12-h light-dark cycle) conditions. Housing and breeding was at the SPF animal facility of the Centro de Estudios Científicos (CECs), Valdivia, which is accredited by the Association for Assessment and Accreditation of Laboratory Animal Care AAALAC. The HA-tagged mouse was generated at the microinjection laboratory at CECs. All animal procedures were approved by the local Institutional Animal Care and Use Committee (protocol N° 2015-01).

### Synthesis of sgRNA, Cas9 mRNA and ssODN Template

A 20 base pair guide (gHA) (5′-ggttttcaggtgggacgtcg-3′) targeting the *Kcnj13* gene region encoding the extracellular loop of the Kir7.1 channel was obtained using Optimized CRISPR software^1^. The synthesis of the chimera gHA-tracrRNA (sgHA) was based on protocols available at Addgene^2^. Briefly, sense and antisense oligonucleotides encoding the guide were synthesized with extra-nucleotides compatible with a digestion product from restriction endonuclease *Bbs*I (5′-CACCGggttttcaggtgggacgtcg-3′ and 5′-AAACcgacgtcccacctgaaaaccC-3′ respectively). Both phosphorylated oligonucleotides were hybridized and cloned in vector pX330 (Addgene 42230) digested with *Bbs*I (Cong et al., 2013). The specific sgHA was obtained by *in vitro* transcription using as a template amplicons of gHA cloned in pX330 vector that include T7 promoter and the reverse primer located downstream of the transcription termination signal (T7sgHA 5′-ttaatacgactcactatagggttttcaggtgggacgtcg-3′ and sgRev 5′-aaaagcaccgactcggtgcc-3′, respectively) (Yang et al., 2013). The 119 bp amplicon was cleaned of RNAses and used for *in vi*tro transcription with the MEGA shortscript T7 Transcription Kit (Ambion, #AM1354) following the supplier indications. The sgRNA was purified by column with MEGAclear Transcription clean up kit (Ambion # AM1908). The Cas9 mRNA was purchased from TriLink BioTechnologies, Inc. (L-6112). The template to repair the Cas9 double strand break and introduce the HA epitope was synthesized as ultramer oligonuclotide DNA single strand (ssODN) in IDT-DNA. The ssODN had 5′ and 3′ homologous arms of 70 bp each (in italics), and a 30 bp core (in boldface) including nucleotides for the HA epitope and *Eco*RV restriction site (ssOD N1620HA: 5′-*gagagaaggagaatgcagctgtgaagctggtgatgtgcttcacacagatgggactagtgtggttttcagg***agcgtagtctgggacgtcgtatggatatcc***gtcgtggtctatttccagatcaccattcatctcagctacagcataccagaggactgcaaagacaagccag*-3′). The edition occurs at position 6210 in the *Kcnj13* gene (Ensembl accession number ENSMUSG00000079436).

### Mouse Zygote Microinjection

Microinjection was performed into the pronucleus and cytoplasm of zygotes obtained by *in vitro* fertilization with sperm and ova from C57BL6 mice. The microinjected mix included sgHA RNA, mRNA Cas9 and ssODN6210HA at 10, 20, and 100 ng/μl respectively. The zygotes were kept in culture until two cell embryo stage and then were transferred to a B6CBA pseudo pregnant female.

### Screening for Genome Modification

Genomic DNA extracted from pup tails were analyzed by PCR and *Eco*RV digestion. The primers flanking the inserted HA epitope region were Kir7.1-HAfor 5′-agtcaaagacaccggagg-3′ and Kir7.1-HArev 5′-tgtgataaaagcctctagc-3′. The expected size amplicons were 420 and 450 bp for wild type and modified allele respectively. The *Eco*RV site incorporated in the modified allele generates 219 and 231 bp products. The right insertion of the HA epitope was confirmed by sequencing the cloned amplicons in pGEM-T vector (Promega).

### Off-Target Analysis

Potential off target genome effects for gHA were obtained using the Optimized CRISPR design software. Specific flanking primers were designed to amplify the potential off targets by PCR. Amplicons were then directly sequenced.

### Cloning of cDNA Kir7.1-HA Channel From Modified Mouse

Total RNA was extracted from an enriched choroid plexus fraction from a heterozygote F1 mouse using Trizol reagent (Life Tech). The cDNA was synthesized using Superscript IV (Invitrogen) and oligo dT primer. The channel open reading frame was amplified by PCR using the Phusion High Fidelity DNA polymerase (Thermo Scientific) and the forward and reverse primers 5′-*aagctt*gcgatggacagcagtaattg-3′ and 5′-*tctaga*tttattctgtcagccctgtttc-3′, including *Hind*III and *Xba*I restrictions sites (in italics) respectively. The amplicons were cloned in pGEM-T easy vector, digested with *Eco*RV to discriminate the modified allele from the wild type and confirmed by sequencing. Finally, the open reading frame was cloned in pCR3.1 expression vector using the *Hind*III and *Xba*I sites to be expressed in HEK-293 cells.

### Heterologous Expression and Electrophysiology

HEK-293 were transiently co-transfected with 0.3 μg of pCR3.1-CD8 and 1.5 μg of plasmid (pCR3.1) containing either Kir7.1wt or Kir7.1-HA cDNA using Lipofectamine 3000 (Invitrogen #L3000). At 24 h post-transfection, the cells were visualized applying Dynabeads-CD8 (Invitrogen #11147D) and recordings were performed from isolated cells using standard whole-cell patch-clamp technique as described elsewhere (Díaz and Sepúlveda, 1995). Currents were recorded using an Axopatch 200B amplifier (Axon Instruments). Acquisition was at 2 kHz with filter at 1kHz employing pClamp10 software (Axon Instruments). For experiments, borosilicate capillaries (Harvard Apparatus GC150F-10, 1.5 mm OD × 1.17 mm ID) were fire polished to 2–4 Mω and covered in beeswax. The bath solution contained in mM: 135 NaCl, 5 KCl, 2 CaCl_2_, 1 MgCl_2_, 1 Glucose, 10 HEPES/Tris, pH7.5 and sucrose to obtain 310 mOsm/L. In alternative bathing solutions NaCl was omitted and KCl or RbCl increased to 140 mM. BaCl_2_ at 1 mM was added to the last solution as indicated. The pipette solution contained in mM: 140 KCl, 1 MgCl_2_, 1 Glucose, 10 EGTA and 10 HEPES/Tris, pH7.5 (300 mOsm/L).

### Choroid Plexus Preparation and Electrophysiology

The choroid plexus was removed from the fourth ventricle of mice killed by decapitation while under deep isoflurane anesthesia (Julio-Kalajzić et al., 2018). The tissue was kept in ice-cold PBS until use (usually within the first 2 h). A small piece of intact tissue was secured to the base of a dish with a platinum wire on the stage of an inverted microscope for studies by patch clamp (Kotera and Brown, 1994). The experiment was similar to that described previously to characterize Kir7.1 activity in retinal pigment epithelial cells (Shimura et al., 2001) and consisted of cell-attached patch-clamp recordings obtained after sealing the pipette to the apical side of the epithelium. The pipette solution contained, in mM: 145 RbCl, 1 MgCl_2_ and 10 HEPES/Tris, pH 7.4, 290 mOsm/L, with or without 1 mM BaCl_2_. For tissue maintenance we used a bath solution containing 140 NaCl, 5 KCl, 2 CaCl_2_, 1 MgCl_2_, 10 Glucose, 10 HEPES/Tris, pH7.4, 310 mOsm/L. For recordings this was exchanged for a high K^+^ solution, chosen to collapse the membrane potential of the cell to zero, in which NaCl was decreased to 5 mM and KCl increased to 140 mM.

### Western Blotting

#### Sample Preparation

Enriched membrane fractions were used for all experiments unless otherwise specified. Three to seven month old mice were euthanized by a lethal dose of anesthesia (Tribromoethanol) and the following tissues were collected: whole eyes, lung lobules, kidney sections and enriched fraction of choroid plexus. Tissues from at least three different animals were analyzed. Trachea epithelium was extracted by scraping and the small intestine epithelia by the protocol described before (Zeineldin and Neufeld, 2012). All tissues were suspended in lysis buffer containing (in mM) 250 sucrose, 20 Tris-Hepes pH 7.4, 1 EDTA, 1 PMFS and protease inhibitor cocktail, and homogenized. Samples were then centrifuged at 4,000 *g* for 15 min at 4°C and the supernatant collected and spun at 17,000 *g* for 30 min at 4°C. The pellet was suspended in lysis buffer, mixed with Laemmli 2X loading buffer, denatured and stored at -80°C until required for use. Proteins were quantified by the Bradford method.

To obtain isolated tubules from kidney papillae, adult mice were anesthetized by intraperitoneal injection of 80 mg/kg ketamine and 10 mg/kg xylazine, and perfused through the aorta with 1ml of incubation solution (0.375 mg/ml glycine, 0.048 mg/ml trypsin inhibitor and 0.025 mg/ml DNAse I in 98b buffer) containing 4 mg/ml collagenase type II. Kidneys were transferred into 98b buffer pH 7.4 of the following composition in mM: 140NaCl, 0.4 KH_2_PO_4_, 1.6K_2_HPO_4_, 1 MgSO_4_, 10Na-Acetate, 1 α-Ketoglutarate, 1.3 Ca-Gluconate. Papillae were dissected and transferred into 1 ml enzyme-containing incubation solution (1 mg/ml) and incubated for 10 min at 37°C (850 rpm). Afterward the supernatant (released tubules) was transferred to a chilled Eppendorf-tube (batch 1) which contains 1ml ice-cold sorting solution (0.5 mg/ml albumin in incubation solution). At the same time 1ml of pre-warmed (37°C) incubation solution was added to the papilla slices to continue shaking for 5 min. The supernatant (released tubules) was transferred again to a chilled Eppendorf-tube (batch 2) containing 1 ml ice-cold sorting solution. This step was repeated to obtain 4 batches. The tubule suspension was gently pipetted into a dissection dish containing 3 ml sorting solution and tubules were selected at 4°C under a stereo microscope. Tubules were transferred directly into Laemmli 2x buffer.

#### Blotting

The samples (10–50 μg of protein) were resolved in SDS-PAGE at 10 or 12% and transferred to nitrocellulose membrane. The membranes were blocked in saline buffer pH 7.4 plus 5% non-fat milk for 1hr at room temperature and incubated with the primary antibody: rat monoclonal anti-HA (1:1000) or rabbit monoclonal anti-HA (1:500) or B1 subunit of V-ATPase (1:500) or AQP1 (1:1000) or actin (1:1000) at 4°C overnight. Then the membranes were incubated with its respective secondary antibody conjugated to HRP, revealed with chemiluminescent HRP substrate and visualized for autoradiography or mini-LAS imaging system (Fuji). Ponceau red or actin was used to check for protein load.

### Immunohistochemistry

Heads of P0 or lungs of adult mice were fixed at 4°C overnight in 4% paraformaldehyde or 10% formalin respectively, then dehydrated in 30% sucrose in phosphate-buffered saline pH7.4 and cryopreserved in Tissue-Tek^TM^ (OCT). Tissues from at least three different animals were analyzed. Cryosections of 8–10 μm were subjected to epitope exposure with 10 mM sodium citrate buffer, pH 6,0. Tissues were treated with 3 or 10% of hydrogen peroxide, blocked with 2.5% normal horse serum, and incubated overnight at 4°C with monoclonal rabbit anti-HA 1:100 or 1:500 for lungs and heads, respectively. Detection of antigen/antibody complexes was performed with ImmPRESS^TM^ reagent kit (Peroxidase) Anti-Rabbit Ig and the reaction was visualized using ImmPACT^TM^ DAB Peroxidase substrate kit. Sections were counterstained with hematoxylin.

### Immunofluorescence

Adult mice were perfused with 4% formalin or 4% paraformaldehyde to isolate brains or lungs, respectively. Tissues from at least three different animals were analyzed. Brains were embedded in OCT and lungs in paraffin. Cryosections (10 μm) were blocked in phosphate saline buffer containing 1% BSA, 0.3% Triton X100 and 10% newborn goat serum and incubated with the monoclonal rat anti-HA (1:1000) overnight at 4°C and then with a secondary antibody donkey anti-rat IgG conjugated with Alexa Fluor 568 (1:2000). All images were captured by confocal microscopy (Olympus FV1000).

### Materials

pX330-U6-Chimeric_BB-CBh-hSpCas9 was a gift from Feng Zhang (Addgene plasmid # 42230). Antibodies against HA-Tag were purchased from Roche, (3F10 clone rat monoclonal) and Cell Signaling (C29F4 Rabbit mAb), Anti β-actin is from Abcam (ab8227). Antibodies against B1 subunit of H-ATPase and AQP1 were a kind gift from Dr. Dennis Brown (Harvard Medical School, Boston, MA, United States) and Sebastian Frische (Aarhus University, Denmark) respectively. Secondary antibodies as ImmPRESS^TM^ reagent kit (Peroxidase) Anti-Rabbit Ig and ImmPRESS^TM^ HRP reagent kit (Peroxidase) goat Anti-Rat IgG mouse adsorbed are from Vector Laboratories, donkey anti-rat IgG conjugated with Alexa Fluor 568 from Thermo Fisher Scientific, goat anti-rabbit IgG and goat anti-rat IgG conjugated to horseradish peroxidase from Bio-Rad and Jackson, respectively. Specifics as ImmPACT^TM^ DAB Peroxidase Substrate Kit from Vector Laboratories, Alexa Fluor^TM^ from Thermo Fisher Scientific, luminol-enhanced chemiluminescence (ECL) from Perkin Elmer Life Science Products and SuperSignal ELISA Femto Maximum Sensitivity Substrate from Thermo Scientific.

## Results and Discussion

To gain an insight into the physiological role of the Kir7.1 channel it is important to precisely determine its expression in different organs and tissues. The use of the optimal negative control, the *Kcnj13* KO mouse lacking the Kir7.1 channel, is not possible as these animals die shortly after birth (Villanueva et al., 2015). An alternative approach is to tag the protein with a suitable epitope using the CRISPR-Cas9 knock-in technology allowing the expression of the channel from its natural chromosomal context. In this case WT animals are a straightforward negative control, provided the tagging does not cause alterations leading to mislocalisation of the protein.

### Strategy to Tag the Kir7.1 Channel

**FIGURE 1:**
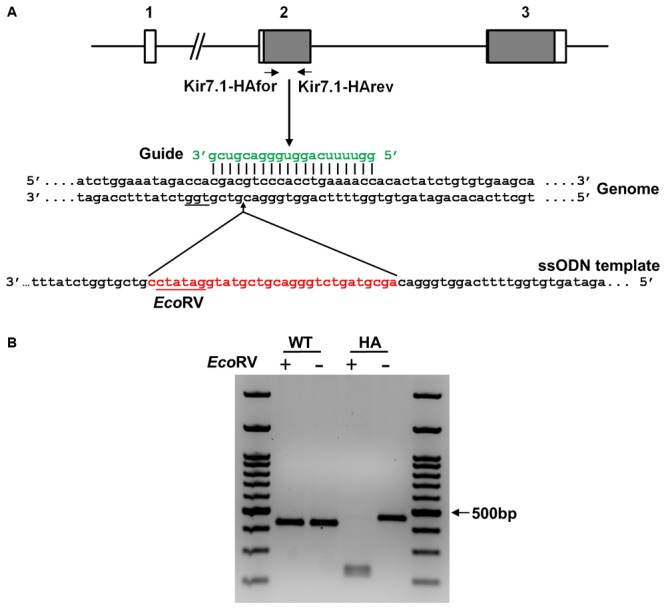
Targeting strategy for the generation of the Kir7.1-HA mice. **(A)** Scheme of the *Kcnj13* gene showing its three exons (boxes) and the coding regions in gray. The guide (green) targets exon 2 in the genome and leads Cas9 to produce a double strand break upstream to the PAM sequence (underlined). The ssODN containing the HA tag sequence, the *Eco*RV site (in red) and the homologous arms (in black) is the template used for homologous directed repair (HDR). **(B)** Agarose gel showing the PCR products of WT and HA genomic DNA obtained using the primers shown in **(A)**. The amplicon digested with *Eco*RV reveals the homozygous (HA) for the tag insertion.

The *Kcnj13* mouse gene is approximately 9.5 kb long, is located in the reverse strand of chromosome 1 and contains 3 exons (**Figure 1A**). The open reading frame of the channel is contained in exons 2 and 3; the N-terminus, both transmembrane domains and the pore domain are encoded in the second exon. The HA epitope was introduced into what is predicted to be an extracellular loop immediately after of the first transmembrane domain. Introduction of such a tag has been demonstrated not to affect the function of the Kir6.1 and Kir1.1 K^+^ channel members of the Kir family (Zerangue et al., 1999; Yoo et al., 2003).

**Figure 1A** also shows schematically the strategy to introduce the HA epitope in the extracellular loop of the channel encoded in exon 2. The guide used to target the specific sequence in the gene is shown in green and the protospacer adjacent motif (PAM) is underlined in the genome sequence. The single strand DNA repair template used to incorporate the HA-coding sequence along with an *Eco*RV site by Homology Directed Repair (HDR) is shown in red. In **Figure 1B**, PCR amplicons from wild type and homozygous littermates for the HA insertion screening can be seen to have the expected size (420 and 450 bp respectively). *Eco*RV cuts only the modified gene yielding 2 restricted fragments (219 and 231bp). Sequencing of the amplicon confirms that the DNA edition occurred at position 6210 of the *Kcnj13* gene (Ensembl accession number ENSMUSG00000079436). Eight potential off target mutations were predicted within gene coding regions and were analyzed by PCR. Direct sequencing of the amplicons did not demonstrate any mutations (results not shown).

### Cloning, Heterologous Expression and Electrophysiological Characterization of Kir7.1-HA cDNA

**FIGURE 2:**
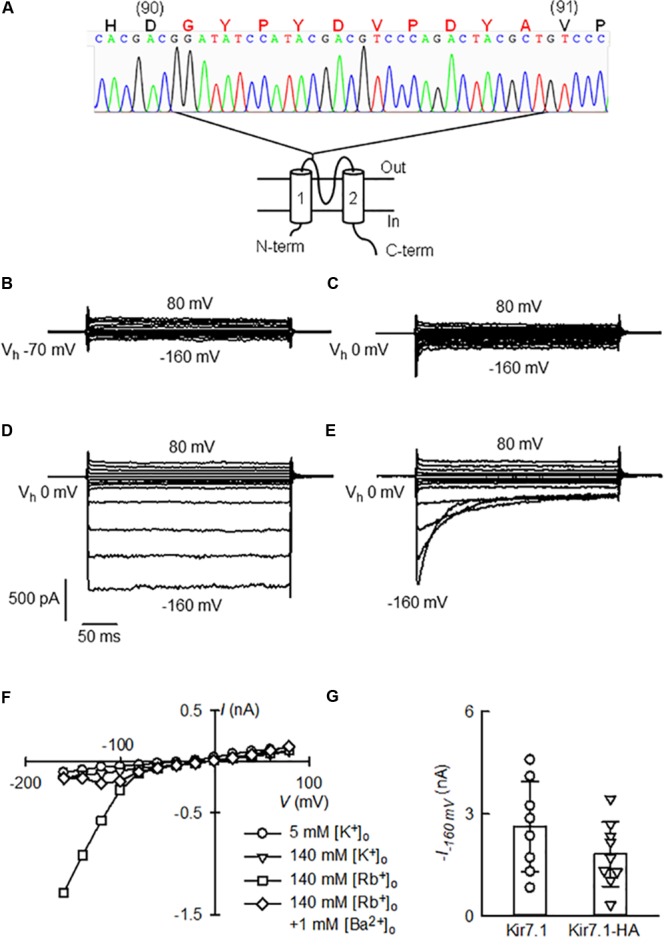
Functional assay of Kir7.1-HA cloned from the choroid plexus of a Kir7.1-HA knock-in mouse. **(A)** Cartoon showing a topological model of the Kir7.1 protein in the membrane and the site of the insertion of the HA-epitope with the chromatogram with of the cDNA sequencing. In capital letters is the amino acid sequence deduced from the nucleotide sequence. Large red letters identify the G plus HA tag (G-YPYDVPDYA) inserted in frame between residues 90 and 91 of the native protein. **(B–E)** Whole-cell currents obtained from a HEK-293 cell after transfection of the Kir7.1-HA cDNA. The cell was held at the stated V_h_ and square voltage pulses were given that took the membrane potential from –160 to 80 mV in 20 mV steps. The cell contained 145 mM K^+^ and the medium was switched from one containing 5 mM K^+^ **(B)** to 145 mM K^+^ **(C)**, 145 mM Rb^+^ **(D)** and finally to 145 mM Rb^+^ with 1 mM Ba^2+^ **(E)**. In **(F)**, current–voltage relations for the currents at the end of the pulses in **(B–E)** are shown. **(G)** Is a comparison of Rb^+^ currents at –160 mV measured in cells transfected with untagged Kir7.1 channel with those of Kir7.1-HA channel (means ± SD and individual points, *n* = 8 and 9 respectively).

We cloned the cDNA of the channel starting from RNA isolated from a heterozygous mouse choroid plexus, a previously described channel expression site (Döring et al., 1998; Nakamura et al., 1999). The chromatogram in **Figure 2A** shows that the HA tag was inserted in frame between residues D90 and V91 and rest of the channel open reading frame was identical to the known cDNA mouse sequence (NCBI Reference Sequence: NM_001110227.2).

To determine whether the modified Kir7.1 protein was transcribed and translated correctly, we examined channel function. The electrophysiological properties of the recombinant Kir7.1-HA channels were studied in transiently transfected HEK-293 cells. Only small currents were seen in 5 or 140 mM extracellular K^+^ solutions (**Figures 2B,C**), but robust inward currents at hyperpolarizing potentials were present when 140 mM extracellular Rb^+^ replaced K^+^ in the extracellular solution (**Figure 2D**). The Rb^+^ inward currents were blocked by the addition of 1 mM Ba^2+^, and this block was strongly dependent on time and membrane potential (**Figure 2E**). **Figure 2F** shows the current-voltage relations taken at the end of the pulses for each of the four extracellular solutions employed. These features are characteristic of native and heterologously expressed Kir7.1 (Wischmeyer et al., 2000; Shimura et al., 2001), suggesting that channel properties are not altered by the HA-tag.

Qualitatively similar currents were also observed in HEK-293 cells transfected with Kir7.1 WT cDNA (not shown). Maximum currents measured in the presence of 145 mM Rb^+^ and at -160 mV (**Figure 2G**), were 2.6 ± 1.33 nA for WT and 1.8 ± 0.94 nA for Kir7.1-HA-expressing cells (means ± SD, *n* = 8 and 9 respectively). WT Kir7.1 currents appeared larger than those generated after transfection of HEK-293 cells with Kir7.1-HA, but this difference did not reach statistical significance (*P* = 0.164 by *t*-test). In conclusion the Kir7.1 channel tagged with the HA-epitope obtained from choroid plexus of knock-in animals behaves very much as the wild type channel.

### Analysis of Kir7.1 Protein Expression by Western Blot

**FIGURE 3:**
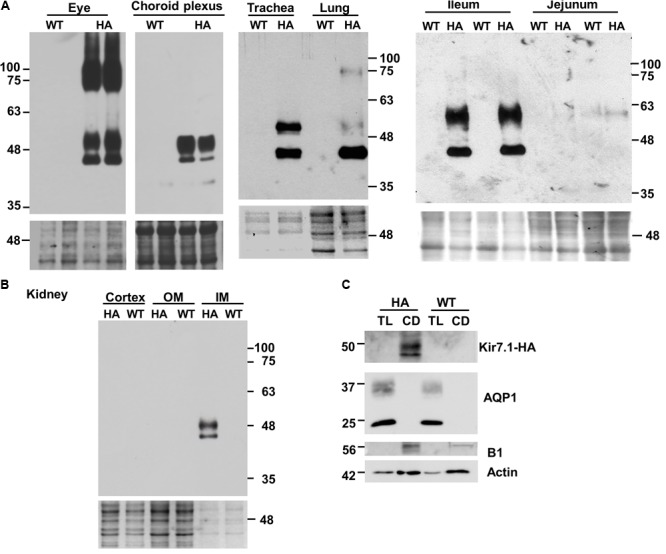
Tissue distribution of Kir7.1-HA protein. Samples obtained from homozygous Kir7.1-HA mice (HA) or wild type mice (WT) were analyzed by western blot. Fractions of membranes obtained from eyes (*n* = 4), choroid plexus (*n* = 4), trachea (*n* = 3), lung (*n* = 10), ileum (*n* = 4), and jejunum (*n* = 4) are shown in **(A)**. In **(B)** kidney plasma membrane fractions of cortex, inner (IM) and outer medulla (OM) are presented (*n* = 3). **(C)** Thin loops of Henle (TL) and collecting ducts (CD) were dissected from renal papillae and analyzed for the presence of aquaporin 1 (AQP1) and proton potassium pump (B1) as markers of TL and CD respectively (*n* = 5). Monoclonal rat anti-HA antibody was used in **(A,B)**, while rabbit anti-HA antibody was used in **(C)**. Ponceau red staining **(A,B)**, or beta actin **(C)** were used as loading controls.

We used anti-HA antibodies to locate Kir7.1-HA expression in different tissues using those from the WT animal as a negative controls. Immunoblots obtained using eye, choroid plexus, trachea and lung, and ileum and jejunum are shown in **Figure 3**. The analysis generally revealed the presence of at least two immunoreactive species in different proportions, one of approximately 45 kD, close to the predicted size of the unglycosylated core of Kir7.1, is present throughout and it appears to be the sole one in the lung. A further species, of around 50 kDa, most likely corresponding to a glycosylated form is more abundant in choroid plexus and renal papilla (panel B) while both species were similarly represented in trachea and the epithelium of the ileum. The expression of the channel is very high in ocular tissue where higher mass bands of 75 and 100 kDa are also present. We have not explored the origin of these species but they might correspond to dimer protein complexes or some degree of protein aggregation. The high amount of Kir7.1-HA in the eye most likely corresponds to the robust expression of the channel in the RPE where it is expressed in the apical membrane of both rat and bovine cells (Kusaka et al., 2001; Yang et al., 2003). The expression in the small intestinal epithelium is consistent with reports showing basolateral membrane expression in non-specified segments of rat small intestine by immunohistochemistry (Nakamura et al., 1999) and human small intestine by Northern blot analysis (Partiseti et al., 1998). Highest, if not exclusive, expression in the mouse is in the ileum. Kir7.1 appeared absent from the jejunum (**Figure 3A**), the large intestine and the stomach (not shown).

**Figure 3B** shows an immunoblot of kidney tissue from Kir7.1-HA and WT animals. Clear bands ascribable to glycosylated and unglycosylated forms are present in tissue from the inner medulla (IM) but are absent from the outer medulla (OM) and cortex. In order to gain more information about where in the papilla the channel is expressed, nephron tubules from collecting duct and thin loop of Henle (TL) were isolated and analyzed for the HA signal and for specific collecting duct and thin loop of Henle markers. As shown in **Figure 3C** the channel signal was present only in collecting ducts (CD), identified by the presence of the B1 subunit of the V-ATPase, but absent from thin limbs of the loop of Henle (TL), where AQP1 signal was present.

Previous work had shown expression of Kir7.1 in rat kidney mainly in the distal convoluted tubule (DCT), connecting tubule (CNT) and cortical collecting duct (CCD), and to a lesser extent in the thick ascending limb of the loop of Henle (TAL), and outer and inner medulla collecting duct (OMCD and IMCD) (Ookata et al., 2000). Results later confirmed for the expression in TAL, DCT and CCD in 21 days old rats (Suzuki et al., 2003). These authors propose a role of Kir7.1 in renal K^+^ excretion under the condition of K^+^ overload. A sharply contrasting distribution was reported for guinea-pig kidney where the main site of expression was judged to be the proximal tubule with some expression also in intercalated cells of the CD (Derst et al., 2001). It was proposed that Kir7.1 is involved in basolateral membrane K^+^ recycling. Our results show that the main site of expression of Kir7.1 is the inner medulla with specific immunoreactivity in the isolated CDs. Unfortunately we could not reliably determine the subcellular localisation of Kir7.1-HA in renal epithelial cells, a failure that we attribute to low level expression. Kir7.1-HA was nevertheless detectable by western blot after pooling sufficient number of isolated tubules. We cannot therefore determine the cell type where the channel is expressed. We speculate that its presence in the inner medulla might have to do with the urine concentration process. An alternative function could be in the acid-base balance process that is sited at the CD intercalated cells. Like other Kir channels Kir7.1 is regulated by intra- and extracellular pH (Hughes and Swaminathan, 2008), which might argue for a role in relation to this last function. This last interpretation could find some support in a recent single cell transcriptomic analysis that reveals high expression of Kir7.1 in intercalated rather than principal cells of mouse kidney CD (Chen et al., 2017).

### Native Kir7.1 Currents in the Choroid Plexus

**FIGURE 4:**
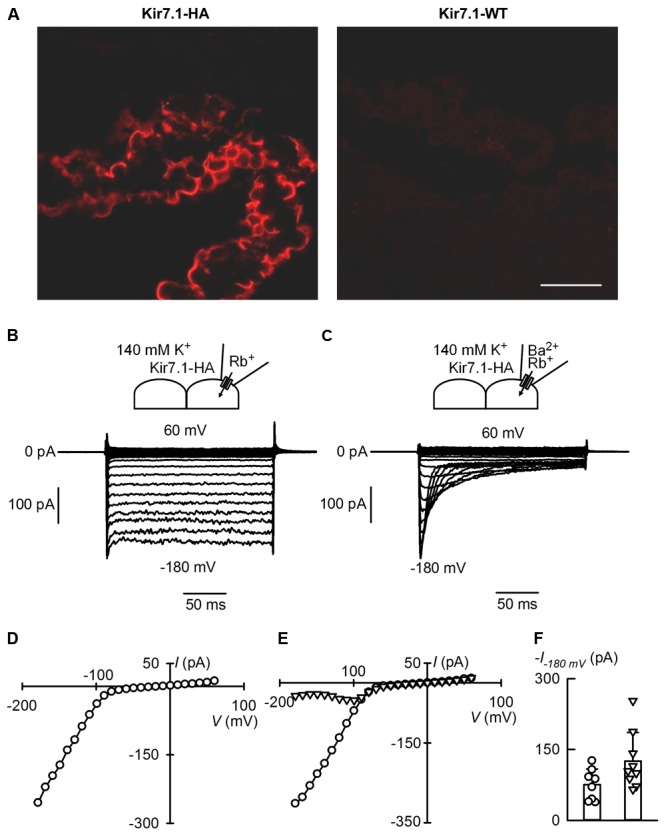
Apical membrane immunofluorescence localisation and ion currents from the choroid plexus of the Kir7.1-HA mouse. **(A)** Cryosections of choroid plexus epithelia were probed with the rat anti-HA monoclonal antibody. Confocal images obtained from choroid plexus of heterozygous Kir7.1-HA (left) and WT littermates WT (right) (*n* = 5). Scale bar represents 50 μm. **(B,C)** Show cell-attached patch-clamp recordings obtained from apical membranes of choroid plexus of Kir7.1-HA mice. The pipette solution contained 145 mM Rb^+^ without **(B)** and with **(C)** 1 mM Ba^2+^. Bath solution had 145 mM K^+^ in both cases. **(D)** Steady-state current-voltage relation for currents measured in **(B)**. **(E)** Current–voltage relations for experiment in **(C)**. Currents shown were taken at the initial peak (circles) or at the end of the pulse (triangles). **(F)** Comparison of maximal currents at –180 mV in recordings from WT (circles and column on the left) or Kir7.1-HA mice (triangles and column on the right). Means ± SD and individual points, *n* = 8 and 9 respectively.

Mildly inwardly rectifying K^+^ currents had been reported in rat choroid plexus (Kotera and Brown, 1994) before molecular identification of Kir7.1. Cloning of Kir7.1 led to its localization in the choroid plexus by *in situ* hybridization and confirmation of the presence of K^+^ currents exhibiting mild inward rectification in whole-cell recordings of rat choroid plexus epithelial cells (Döring et al., 1998). Immunolocalization, on the other hand, demonstrated Kir7.1 immunoreactivity at the apical membrane of rat choroid plexus epithelium (Nakamura et al., 1999). The western blots of choroid plexus proteins in Kir7.1-HA mouse tissues in **Figure 3A** are consistent with high expression of the channel in this epithelium. To examine whether Kir7.1-HA channels are sorted properly and able to mediate currents in choroid plexus epithelial cells we explored their presence by immunofluorescence and assayed for their currents by patch-clamp. The immunofluorescence against HA tag confirms that the protein is located at the apical membrane of choroid plexus epithelium from Kir7.1-HA. There was no HA-immunoreactivity in tissue from WT mice (**Figure 4A**). To assay for Kir7.1 mediated currents we used an approach successfully employed previously in RPE cells to measure Rb^+^ inward currents in cell-attached patches of membrane (Shimura et al., 2001). Because the conductance of Kir7.1 to Rb^+^ is ∼10 times higher than that for K^+^ (Wischmeyer et al., 2000), using this cation could allow the detection of Kir7.1-mediated inward currents across choroid plexus apical membranes in cell-attached configuration if there is a high density of channel proteins. **Figure 4B** shows a cell-attached recording from choroid plexus apical membrane of a Kir7.1-HA mouse obtained with a pipette containing 145 mM Rb^+^. Inward currents were evident at potentials more negative than -60 mV (**Figure 4D**). Similar experiments but with a pipette solution containing 1 mM Ba^2+^ in addition to 145 mM Rb^+^ yielded the type of currents shown in **Figure 4C**. Initially currents were inwardly rectifying but a time-dependent inhibition took place at the most hyperpolarized potentials. Initial (circles) and steady-state (triangles) currents are plotted as function of voltage in **Figure 4E** revealing the membrane potential-dependent block of Rb^+^ influx quite characteristic of Kir7.1. Rb^+^ currents assayed in plexuses from Kir7.1-HA knock-in mice behaved similarly to those from WT mice (not shown). A comparison of the initial Rb^+^ currents at -180 mV in choroid plexuses from Kir7.1-HA (circles) and WT mice is shown in **Figure 4F**. No difference between choroid plexus HA-tagged and WT Kir7.1 currents was observed (*P* = 0.0584 by unpaired *t*-test).

The choroid plexus epithelium, together with RPE, must be a site of very high expression of Kir7.1. This is suggested by the strong signal seen both in western blots and in immunofluorescence assays. More importantly the large currents carried by Rb^+^ in cell-attached patches also suggest a very high channel expression in the apical membranes of this epithelium and of the same order as those reported in the RPE (Shimura et al., 2001). Kir7.1 (and Kv1) channels are thought to play a key role in the secretion of cerebrospinal fluid (CSF) by choroid plexuses (Damkier et al., 2013). They provide an essential “leak pathway” for K^+^ pumped into the cells by the Na^+^-K^+^ ATPase which is also very highly expressed in the apical membrane of the choroid plexus and is critical in the process of CSF secretion (Damkier et al., 2013). As reviewed by Damkier et al. (2013), the Na^+^-K^+^ ATPase transports significant amounts of K^+^ from the CSF into the choroid plexus cells. In addition it sets up the gradients for effective functioning of the NKCC1 of the apical membrane, an exporter of K^+^ from the CSF. The close association of Kir7.1 with the Na^+^-K^+^ ATPase suggests a functional coupling that has been speculated to be necessary to ensure efficient function of the pump (Hibino et al., 2010). Most of the ion transporters involved both in the maintenance of a low CSF K^+^ concentration and those involved in the ion transport processes that sustain fluid secretion are secondary active transport mechanisms dependent upon the gradients set up by the pump. If this hypothesis holds true, Kir7.1 would be necessary for efficient pump function and would therefore be a major contributor to the low CSF K^+^ concentration and CSF production. Modulation of Kir7.1 activity may therefore be important in controlling the rate of CSF secretion (and hence intracranial pressure), and in maintaining a stable [K^+^]_CSF_, which is a requirement for the central nervous system function.

### Distribution of the Kir7.1 Channel in the Respiratory Tract Epithelium

**FIGURE 5:**
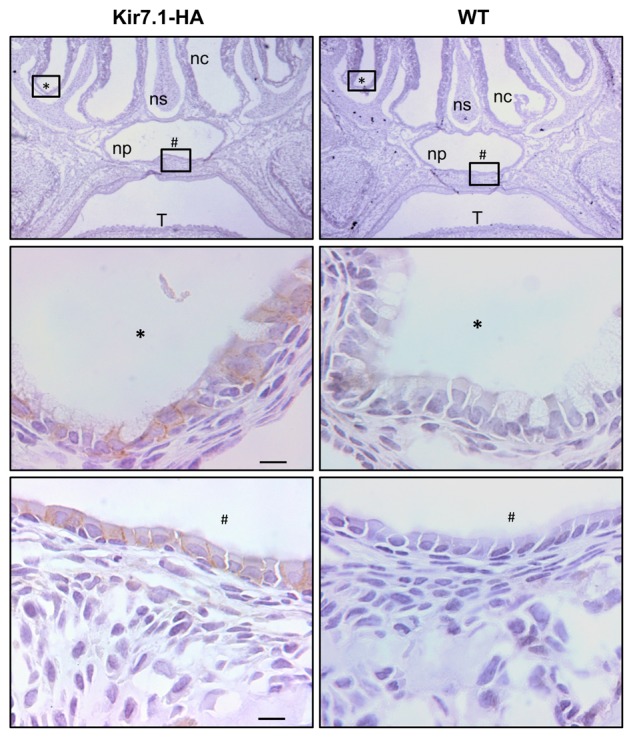
Kir7.1-HA channel is located in the basolateral membrane of the nasopharyngeal respiratory epithelium. The immunohistochemically analysis from Kir7.1-HA (left) and Kir7.1-WT (right) newborn mice tissue was performed with the rabbit anti-HA antibody (*n* = 4). In the (middle) and (bottom) panels amplifications of marked regions (^∗^) and (#) are shown. Counterstaining was performed with hematoxylin. Scale bar represents 10 μm. ns, nasal septum; nc, nasal cavity; np, nasopharynx; T, tongue.

In our previous work using the LacZ reporter gene we showed that the promoter of *Kcnj13* gene is active in embryos and newborn mice in the upper airway epithelium (Villanueva et al., 2015). Here we use the HA immunoreactivity of Kir7.1-HA tissues to determine the protein localization at P0. As shown in the **Figure 5** (see two upper panels) the development of the palate, as expected, is completely normal in the KI mouse unlike what occurs in the *Kcjn13* null mutant mouse that presents complete secondary cleft palate. As it is shown in left hand side panels of **Figure 5**, the channel is expressed only in the basolateral membrane of respiratory epithelial cells in the nasal cavity and nasopharynx in the Kir7.1-HA mice. We have demonstrated using the lacZ reporter in heterozygous KO mice, which present normal palate development, that the channel is expressed only in the epithelial cells covering the nasal aspect of the palatal shelves (Villanueva et al., 2015). This agrees with present results as no Kir7.1-HA signal was detected in the epithelium of the oral cavity side of the palate. As shown in right hand side panels of **Figure 5**, the immunoreactivity is completely absent from tissues of WT littermates.

**FIGURE 6:**
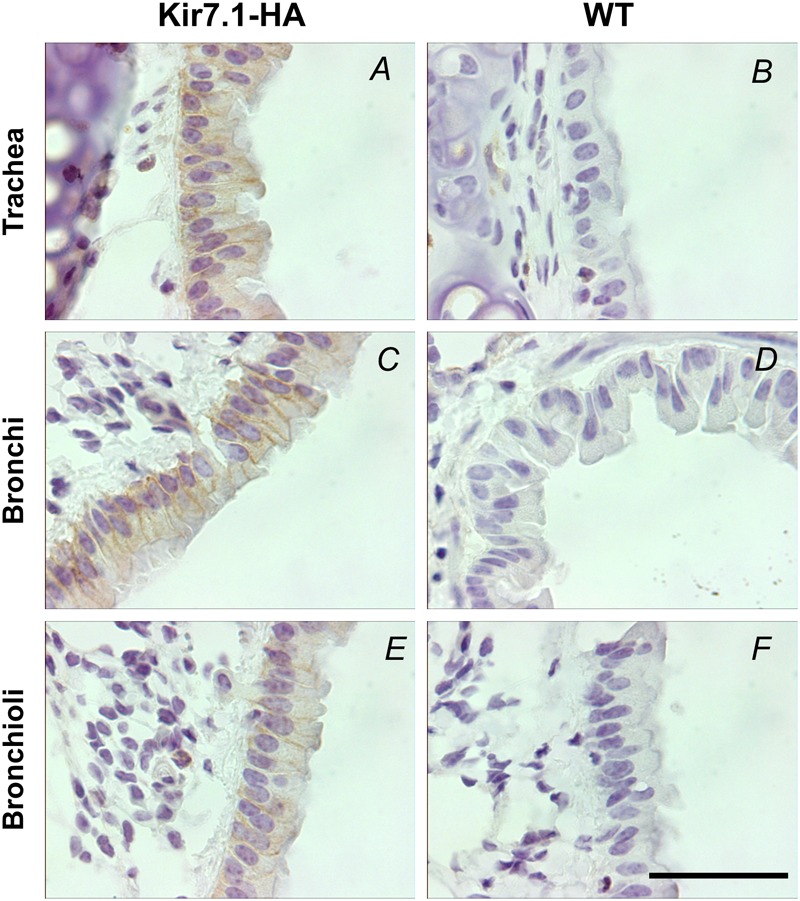
Kir7.1-HA channel is located in the basolateral membrane in respiratory epithelium of adult mice. The detection of HA tag was performed in trachea, bronchi and bronchioli immunohistochemically with the rabbit anti-HA antibody (*n* = 3). **(A,C,E)** Are samples from Kir7.1-HA mice and **(B,D,F)** from WT mice. Scale bar represents 50 μm.

We then studied the expression of the channel in the epithelial cells of lower airway epithelia in adult mice. We first performed an immunohistochemistry assay with a rabbit monoclonal anti-HA antibody. The analysis revealed the expression of Kir7.1-HA in the basolateral membranes of the epithelial cells from trachea, bronchi and bronchioli (**Figures 6A,C,E**). The staining for Kir7.1-HA was absent from WT tissues (**Figures 6B,D,F**).

The impression of a basolateral expression for Kir7.1 gained from our previous immunolocalisation studies performed using an anti-Kir7.1 antibody (Villanueva et al., 2015) is confirmed here using HA immunoreactivity with appropriate negative controls. Our previous work has also shown a moderate retardation in lung development in *Kcnj13* null mutant embryos and we speculated that this might have due to a deficiency in fluid secretion secondary to channel deficiency. Electrolyte transport is involved in the maintenance of the airway fluid layer covering the whole of the respiratory tract that is essential to its normal function including mucociliary clearance. Processes include absorptive and secretory functions that require the activity of basolateral Na^+^/K^+^ pump. K^+^ channels are required to avoid intracellular accumulation and to stabilize the membrane potential favorable to Na^+^ absorption and or Cl^-^ secretion (Hollenhorst et al., 2011; Lee and Foskett, 2014).

## Conclusion

Genetically modified mice are useful in unraveling the function of ion channels *in vivo*. We have been interested in understanding the function of Kir7.1 in the various organs where it is expressed. Knockout mice are valuable in ascertaining the function of ion channels and, importantly, they aid in the cellular and subcellular localization by providing reliable negative controls for western blots and immunolocalisation assays. We previously generated a mouse deficient in the channel by ablation of the *Kcnj13* gene, but unfortunately total channel inactivation leads to neonatal mortality precluding its further use (Villanueva et al., 2015). The generation by CRISPR Cas9 technology of a Kir7.1 knock-in mouse that expresses the channel tagged with an HA provides a convenient animal model to study reliably the distribution of the channel in mouse tissues using their WT littermates as negative controls. We here confirm the localization of Kir7.1 in the eye and choroid plexus, where it is present at high levels. We also confirm the intestinal localization of Kir7.1 and we identify the ileum as the exclusive or main site of expression. We show that Kir7.1 is mainly expressed in the renal papilla where it is present in IMCD isolated tubules. This contrasts with previously reported wide distribution in rat kidney and proximal tubule expression in guinea-pig nephron. We also confirm our previous suggestion of a basolateral expression in the respiratory tract epithelium from the trachea to the bronchioli. We also found similar basolateral membrane localization in the epithelium covering the nasal cavity and nasopharynx in newborn animals which has not been previously reported. Functional assays demonstrate that Kir7.1-HA cloned from the knock-in animal and expressed heterologously has unaltered electrophysiological properties attesting to the lack of effect of the epitope insertion in channel function. In a similar manner, the native current recorded from choroid plexus demonstrates the normal function of the channel *in situ*, incidentally revealing the very high expression of Kir7.1 in this tissue and suggesting it might play an important role in CSF production. Recent evidence for ion channel function independent of their conductive properties such as in signaling complexes termed chanzymes and chansporters (Krapivinsky et al., 2014; Abbott, 2017) could be advantageously studied using the modified channel knockin approach described here. Additionally the tagged channel approach could be used in investigating the expression of the channel during development. In summary the Kir7.1-HA knock-in mouse characterized in the present work could become, perhaps together with complementary tissue specific knockout models, a useful tool to unravel Kir7.1 function.

## Author Contributions

The experiments were performed at the Centro de Estudios Científicos (CECs) Biological Laboratory. IC, RC, MN, PB, FS, and LC conceived and designed the work. IC, SV, JB, KL-C, FJ-K, NB, MN, DF-F, and PB, performed the experiments and together with LC and FS analyzed and interpreted the data. IC, FS, and LC drafted the work that was additionally revised critically by all authors. All authors approved the final version of the manuscript and all those that qualify for authorship are listed.

## Conflict of Interest Statement

The authors declare that the research was conducted in the absence of any commercial or financial relationships that could be construed as a potential conflict of interest.

## Footnotes

1 http://crispr.mit.edu/

2 https://www.addgene.org/crispr/zhang/
